# A heading date QTL, *qHD7.2*, from wild rice (*Oryza rufipogon*) delays flowering and shortens panicle length under long-day conditions

**DOI:** 10.1038/s41598-018-21330-z

**Published:** 2018-02-13

**Authors:** Li Jing, Xu Rui, Wang Chunchao, Qi Lan, Zheng Xiaoming, Wang Wensheng, Ding Yingbin, Zhang Lizhen, Wang Yanyan, Cheng Yunlian, Zhang Lifang, Qiao Weihua, Yang Qingwen

**Affiliations:** Institute of Crop Science, Chinese Academy of Agricultural Sciences, Beijing, 100081 China

## Abstract

Heading date (HD) and panicle length (PL) are important traits that affect rice breeding and are controlled by pleiotropic genes. Some alleles associated with HD and PL from wild relatives might differ from those in cultivated rice. In this study, a main effect HD quantitative trait locus from wild rice, *qHD7.2*, was identified using a chromosomal segment substitution line (CSSL) population. First, *qHD7.2* was determined to be located near RM172 on chromosome 7 based on association analysis of phenotype data from six environments and 181 polymorphic molecular markers. CSSL39, which has the latest flowering of all CSSLs and carries *qHD7.2*, was selected for further study, and *qHD7.2* was narrowed to a 101.1-kb interval using a CSSL39/9311 F_2_ population. An *OsPRR37*-homologous gene was found within this region. The wild type allele delayed flowering and shortened PL under long-day conditions. The *HD7.2*, which was identified as a candidate gene for *qHD7.2*, transcript level was substantially higher than that in 9311. Our data showed that *HD7.2* is likely a novel *OsPRR37* allele. Sequence analysis revealed that *OsPRR37* in cultivated rice had multiple origins, and natural variation in the coding domain sequence and promoter region contribute to flowering time diversity in cultivated rice.

## Introduction

Asian cultivated rice varieties are distributed worldwide in a wide range of latitudes, from 53° north to 40° south; alternatively, its direct ancestor, common wild rice (*Oryza rufipogon*), is a short-day plant^1^. Artificial selection from naturally variable photoperiod sensitivity traits in rice germplasm is of paramount importance for rice global adaptation^2^. Heading date (HD) is a crucial determinant of rice diversification and domestication^3,4^. HD in rice is a typical quantitative trait locus (QTL) with complex inheritance, and is controlled by multiple genes. Clearly understanding the genetic basis of HD is important for elucidating the adaptation of rice to different cultivation areas and crop seasons.

Over the last two decades, hundreds of HD QTLs were documented in the Gramene database^5^ (http://archive.gramene.org/qtl/). Almost 65 HD genes have been cloned, and the photoperiod regulatory network are well documented. *Hd3a* (a rice orthologue of the *Arabidopsis FT* gene) and *RFT1* (the closest homologue to *Hd3a*) are two florigen genes in the complex flowering time control network^6,7^, and the flowering-integrated factors *Hd1* and *Ehd1* receive signals from other genes to regulate flowering by affecting florigen gene expression^8,9^. Of these other genes, *OsELF3*^10^, *OsGI*^11^, *SE5*^12^, *PhyB*^13^, and *Hd6*^14^ regulate the expression of *Ghd7*^15^, *Ehd3*^16^, *OsLFL1*^17^, *Hd16/EL1*^18^, *RID1*^19^, and *OsMADS51*^20^ and belong to the *Ehd1*-dependent pathway. Additionally, several HD genes such as *OsPRR37*^21^, *OsCO3*^22^, *DTH2*^23^, and *OsDof12*^24^ directly control the expression of florigen genes independent of *Hd1* and *Ehd1*. Molecular analysis revealed that natural variation in coding domain sequence and promoter sequences of some HD-QTL genes led to different responses to photoperiod sensitivity. Takahashi^25^ found that diverse combinations of natural variation in Hd1 proteins, *Hd3a* promoters, and *Ehd1* expression levels contribute to flowering time diversity in the core collection of rice cultivars. Panicle length (PL) is an important trait which strongly affects yield components by determining panicle architecture in rice, such as grain number per panicle. PL can be used as a selection criterion for yield breeding and exhibits higher heritability than yield itself ^26^. Several QTLs associated with PL have been reported; among them, only *dep1* and *sp1* that lead to shorter panicles have been cloned^27,28^.

As an increasing number of HD genes have been identified, the association of HD and PL has been identified in pleiotropic genes. Both HD and PL are key factors that influence the business value of cultivated rice. It has been reported that PL is co-segregated and finely mapped with the HD locus, which demonstrates the pleiotropic effects of the underlying genes^29,30^. *Hd1*^29^, *Ghd7*^15^, and *Ghd8*^30^ are pleiotropic, and prolong HD and enhance PL. However, genetic factors that affect HD and their effects on PL are not well understood. Moreover, a large of proportion of these QTLs have still not been finely mapped or cloned, and more HD genes are needed to elucidate the associated genetic interactions^31,32^.

Common wild rice (*Oryza rufipogon Griff*.) evolved many exotic genes in response to a variety of disasters and natural selection of harsh environments, and exhibits a late HD and open panicle architecture compared with cultivated rice^33^. Chromosome segments substitution lines (CSSLs) greatly improve the accuracy of gene or QTL mapping by eliminating the influence of genetic backgrounds. Thus, CSSLs have been used to obtain many cloned and fine-mapped QTLs, because they represent ideal material for genetic analysis and gene fine mapping^14,34–39^. Numbers of QTLs from wild rice species were reported and some alleles increase PL^40,41^.

*OsPRR37*, which is responsible for the *Hd2/qDTH7-2* QTL, was reported as a major effect QTL that control photoperiod sensitivity in rice^42^. The genes homologous to *OsPRR37* in barley, wheat, and sorghum have been well studied with regard to how they impact photoperiod sensitivity^43–45^. Natural variants of *OsPRR37* have also been reported that contributed to the expansion of rice cultivation to temperate and cooler regions^21^. However, the natural variants of promoter of *OsPRR37* have remained elusive.

To explore new allele from wild rice, we constructed a set of wild rice CSSLs in the previous study. We herein report the detection and fine mapping of a QTL for HD and PL. In this study, we performed QTL analysis for HD and PL using CSSLs and advanced backcross populations. One QTL from wild rice, *qHD7.2*, was identified and a novel allele of *OsPRR37* was fine mapped within this QTL. Expression pattern of this gene was analyzed between wild and cultivated rice, and sequence analysis among 3000 germplasm was performed to identify the origin and selection domain during rice domestication. Our study provides a new genetic resource for cultivated rice breeding and new evidence regarding the evolution of flowering genes.

## Results

### *qHD7.2* detection using a CSSL population

**Figure 1:**
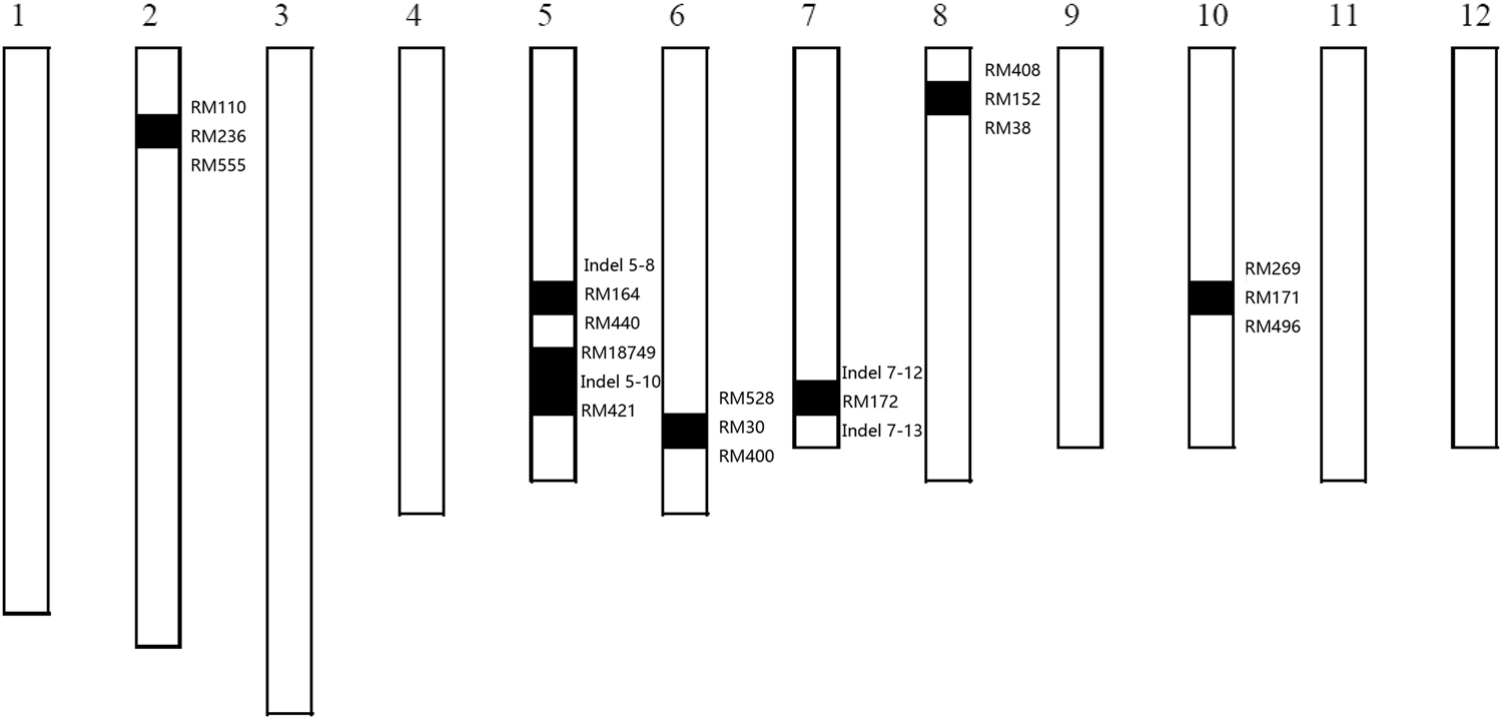
Graphical genotypes of CSSL39. Black bars indicate chromosomes of wild rice. The number of Chromosome was marked above each chromosome, the SSR markers were showed at the right of each substitution segment.

In our previous study, a set of 198 CSSLs population were developed from common wild rice as the donor parent and an elite *O.sativa indica* cultivar, 9311 as recurrent parent^46^. The HD and PL were remarkably different between the two parents; the wild rice has an open panicle phenotype and does not undergo heading under long-day conditions. The HD phenotype substantially differed within the CSSL population. The days to heading we obtained from six environments (two years, three sites) were associated with genotype based on 119 simple sequence repeat (SSR) and 62 insert/delete (InDel) polymorphic markers evenly distributed across the 12 rice chromosomes in our laboratory, several HD QTLs were identified based on a cut-off LOD score ⩾ 2.5 (Table 1). Two QTLs located, one located near RM172 on chromosome (Chr.) 7 and one located near RM19 on Chr. 12, were detected in all environments. These loci showed an increasing effect on HD; this indicates that these two QTLs were stably detected, and they could improve accuracy and efficiency of future work. In particular, the QTL near RM172 identified under the Beijing conditions had the highest LOD value (14.6) and explained 27.2% of the variance, which indicated that this QTL (named *qHD7*.2) is likely a main effect QTL. One CSSL, CSSL39, which flowers the latest of all CSSLs under natural long-day (NLD) conditions in Beijing (116.4°E/39.9°N, day length > 15 h) and carries the *qHD7*.2, was selected for advanced study. The CSSL39 genotypes were shown in Fig. 1. Seven substituted segments from wild rice were detected in the whole CSSL39 genome (Fig. 1). The markers associated with substituted segments included RM236 on Chr. 2; RM164, Indel 5-10 on Chr. 5; RM30 on Chr. 6; RM172 on Chr. 7; RM152 on Chr. 8; and RM171 on Chr. 10.

**Table 1:** Heading date QTLs detected in the 198 CSSLs from three environments.

Marker	Chr.	Environment	LOD value	PVE(%)	Add
RM172	7	Beijing	14.58	27.2	5.018
		Nanjing	6.96	9.997	2.872
		Sanya	2.669	6.708	1.895
RM19	12	Beijing	4.001	5.987	3.044
		Nanjing	5.528	7.301	3.173
		Sanya	4.134	11.36	3.188
RM590	10	Nanjing	2.86	15.3	21.2
		Sanya	2.12	10.2	5.6
RM523	3	Sanya	4.921	11.09	−2.586
RM411	3	Sanya	4.122	9.459	−2.643

The data for each environment represent data averages over two years. LOD indicates LOD score calculated from single marker analysis. PVE(%) represents phenotypic variation explained by the marker. Add indicates estimated additive effect of the marker.

### Phenotypic characterisation of CSSL39, 9311, and the F1 generation of CSSL39/9311

**Figure 2:**
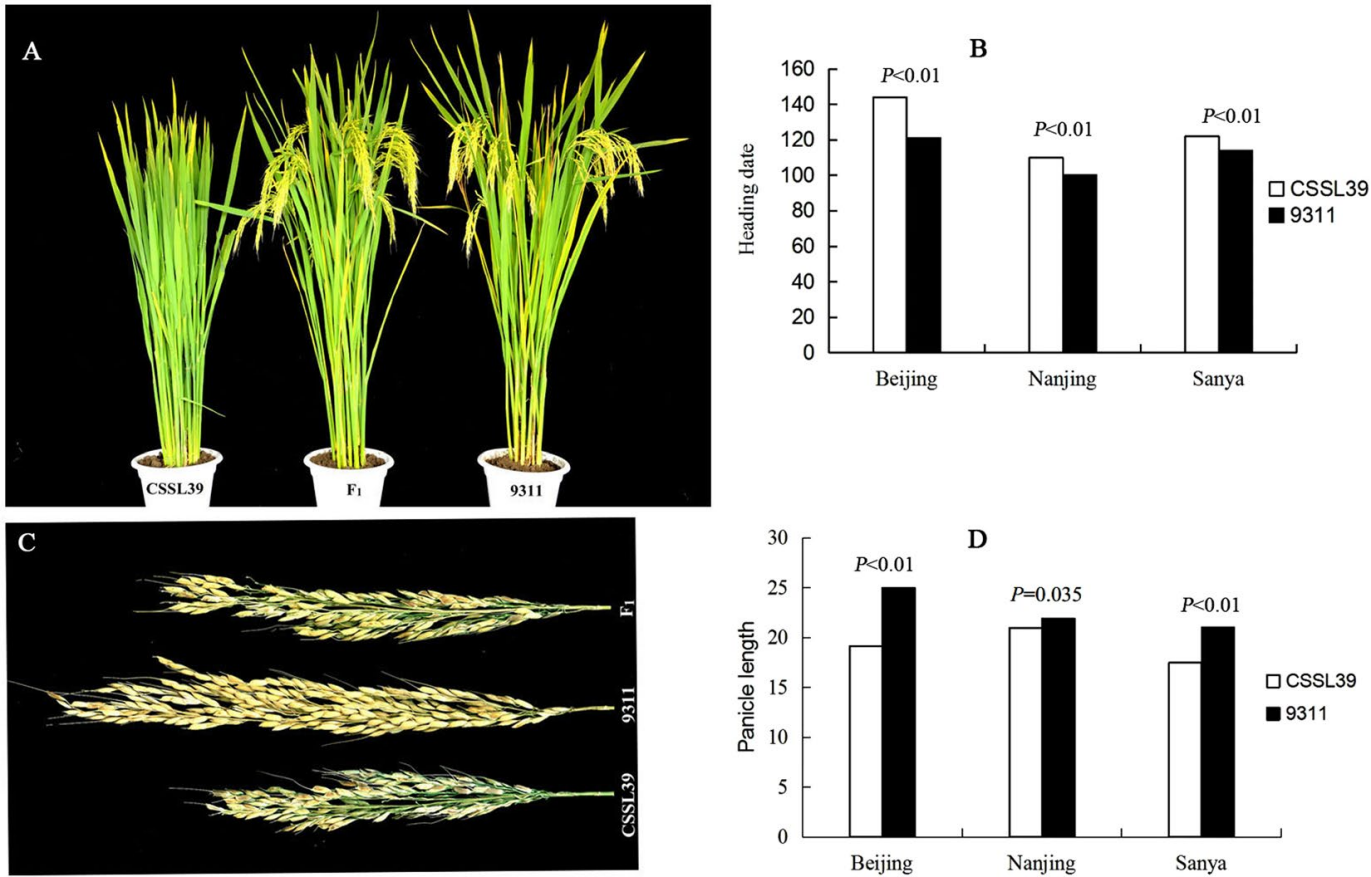
Phenotypic characterisation of CSSL39, 9311, and the F1 generation of CSSL39/9311. (**A**) Heading date phenotypes of CSSL39, F_1_, and 9311. Photos were taken under natural long-day (NLD) conditions in Beijing. (**B**) Heading date comparison between CSSL39 and 9311 in three environments. (**C**) Panicle length phenotypes of CSSL39, 9311, and F_1_ under NLD conditions in Beijing. (**D**) Panicle length comparison between CSSL39 and 9311 in three environments. P values were obtained in two-tailed t tests.

Compared with the recurrent parent 9311, CSSL39 exhibited a 24-d delay under NLD conditions in Beijing, 14-d delay in Nanjing, and 7-d delay under natural short-day (NSD) conditions in Sanya (110.0°E/18.5°N, day length <12 h), and the HD of F_1_ progeny fell in between those of the two parents (Fig. 2A,B). Additionally, we investigated CSSL39 basic agronomic traits, including thousand grain weight, grain length, grain width, plant height, flag leaf length, flag leaf width, PL, and tiller number (S-Table 1). We found that only PL was significantly shorter compared with 9311; CSSL39 PL was 5.88 cm shorter under NLD conditions and 3.58 cm shorter under NSD conditions compared with that of 9311, the F_1_ generation also exhibited a moderate PL (Fig. 2C,D). Moreover, we counted of the number of mature CSSL39 and 9311 seeds based on seed coat colour; the results showed that approximately 88.9% of 9311 grains but only 25% of CSSL39 grains reached maturity under NLD conditions. However, there were no significant differences in maturity between CSSL39 and 9311 under NSD conditions (S-Fig. 1), which indicated that 9311 is reproductively better adapted to the NLD conditions.

### Genetic analysis and fine mapping of *qHD7.2* using the CSSL39/9311 F_2_ population

**Figure 3:**
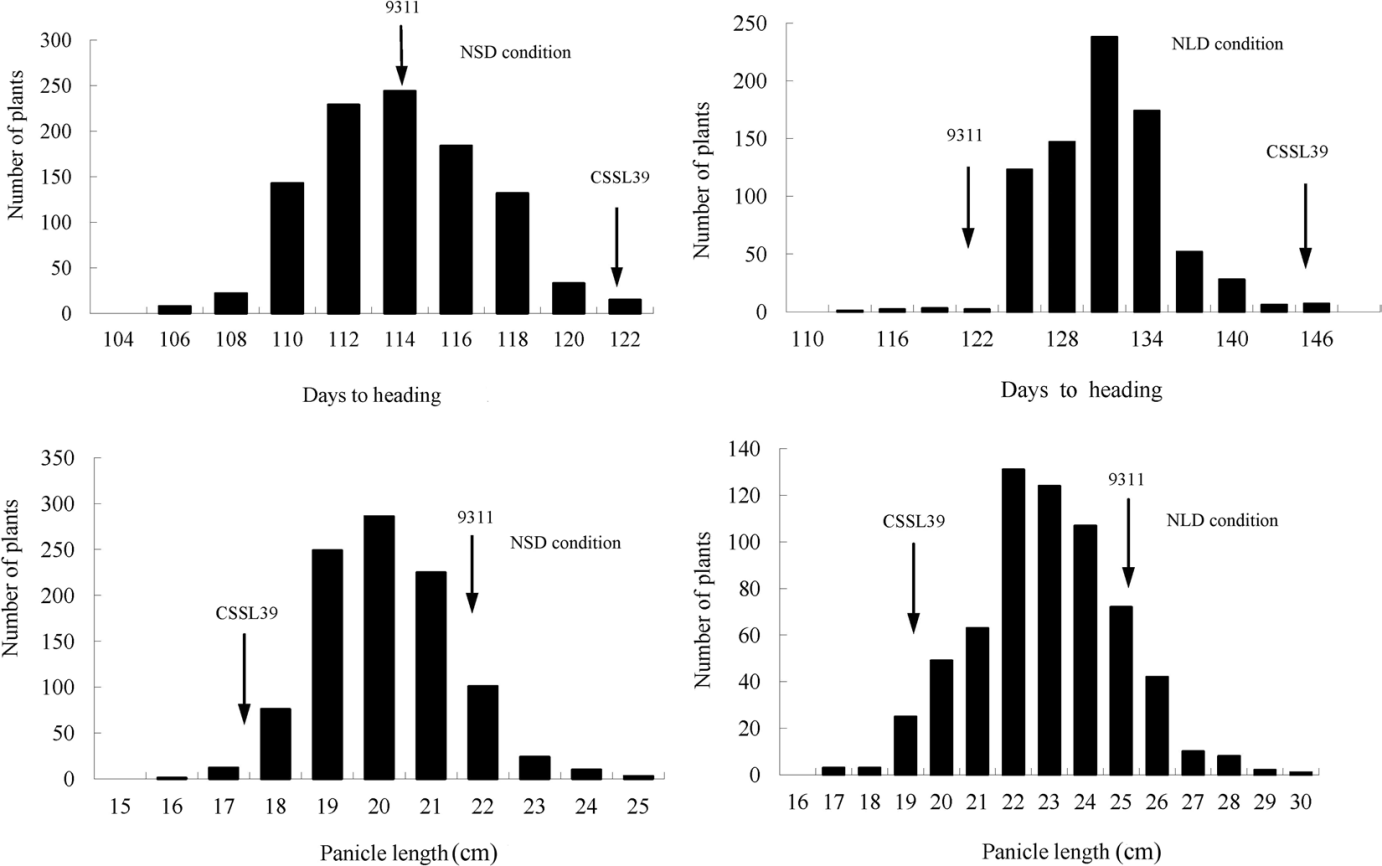
Frequency distribution of heading date and panicle length in CSSL39/9311 F_2_ populations under two photoperiodic conditions (NLD and NSD). Black arrows show average heading dates and panicle lengths of CSSL39 and 9311.

An F_2_ population of CSSL39/9311 was constructed for genetic analysis and fine mapping of *qHD7.2*. The frequency distribution for HD and PL in the F_2_ segregating generation exhibited a typical normal distribution. A wide range of phenotypes presented, from 105 d to 122 d for HD and 17.2 cm to 24.7 cm for PL of 1024 individuals from Sanya, and 112 d to 146 d for HD and 16.6 cm to 29.4 cm for PL of 846 individuals from Beijing. For HD and PL, most of the F_2_ segregating individuals are midway between those of the two parents. Meanwhile, the existence of two-way transgressive separation was found in PL (Fig. 3).

**Figure 4:**
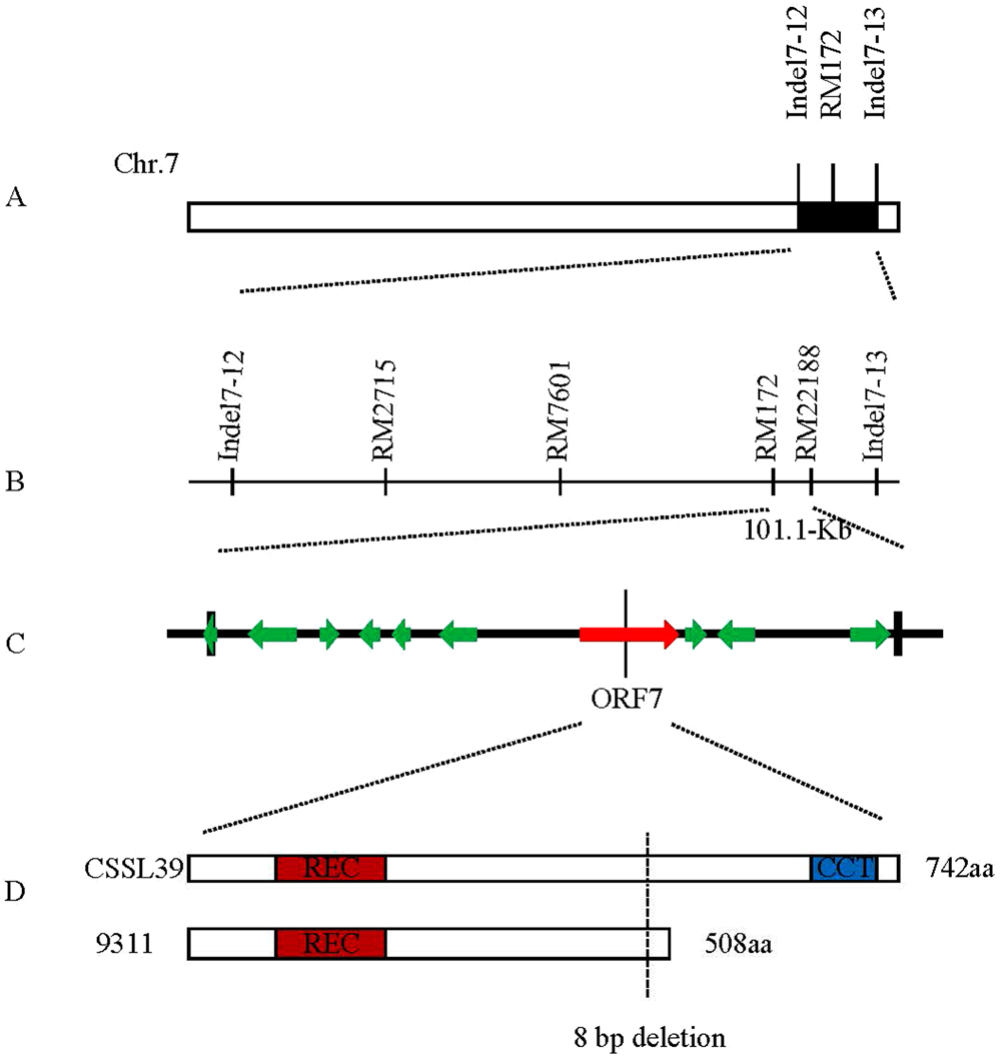
Fine mapping of *qHD7.2*. (**A**) The *qHD7.2* locus was mapped between the markers Indel7–12 and Indel7–13 on chromosome 7. (**B**) Fine mapping of *qHD7.2* using an F_2_ secondary segregation population. *qHD7.2* was determined to belong to a 101.1-kb genomic region between the markers RM172 and RM22188. (**C**) Ten ORFs were identified between the RM172 and RM22188 markers. (**D**) Candidate gene ORF7 protein structure in CSSL39 and 9311. REC indicates signal receiver domain, CCT indicates CCT domain, and aa indicates amino acids.

Sixteen new InDel and SSR markers located at the substituted interval near RM172 were used to screen the parents, CSSL39 and 9311, of which 10 markers exhibited polymorphism (S-Table 2). First, the substituted segment near RM172 was narrowed to the interval between InDel7–12 to InDel7–13 (Fig. 4A). Combined with the eight SSR markers which were detected in CSSL39, we used these 18 polymorphic markers for CSSL39/9311 F_2_ population screening. HD and PL QTLs were detected in 1024 F_2_ individuals from Sanya and 846 F_2_ individuals from Beijing. In total, two HD and three PL QTLs were detected (Table 2). Two HD QTLs (*qHD7.1* and *qHD7.2*), which can be stably inherited, were detected under both NLD and NSD conditions. *qHD7.1* was mapped in the 522.9-kb interval of RM7601 to RM172 on Chr. 7, whereas *qHD7.2* was mapped in the 101.1-kb interval of RM172 to RM22188 (Fig. 4B). They maintained the same genetic effects that prolong HD. Three PL QTLs (*qPL7*, *qPL10.1*, and *qPL10.2*) were detected. *qPL7* was mapped in the 101.1-kb interval of RM172 to RM22188 at the same position of *qHD7.2*. *qPL10.1* was mapped in the 568-kb interval of RM171 to RM1146 on Chr. 10, and *qPL10.2* was mapped in the 69.7-kb interval of RM25723 to RM7300 on Chr. 10. Among those three QTLs, *qPL10.1* was only detected under NSD conditions; *qPL7* and *qPL10.2* can be stably inherited under both NLD and NSD conditions. Only *qPL10.2* under NSD conditions had a positive effect on elongating the panicle; the others had negative effects on shortening the panicle. Therefore, we predicted that *qHD7.2* is likely a major QTL for delaying HD and shortening PL.

**Table 2:** Genetic effects of heading date and panicle length QTLs estimated under NSD and NLD conditions.

QTL	trait	Chr.	Intervals	Hainan(Winter, 2015, SD)				Beijing(Summer, 2015, LD			
				LOD	PVE(%)	Add	Dom	LOD	PVE(%)	Add	Dom
*qHD7.1*	HD	7	RM7601-RM172	39.69	46.65	2.855	−0.464	17.08	35.19	4.597	1.158
*qHD7.2*	HD	7	RM172-RM22188	39.71	46.30	2.844	−0.47	15.34	32.38	4.334	1.805
*qPL7*	PL	7	RM172-RM22188	7.156	6.748	−0.466	−0.017	7.981	16.97	−1.038	0.067
*qPL10.1*	PL	10	RM171-RM1146	8.414	22.08	−0.366	−1.239	—	—	—	—
*qPL10.2*	PL	10	RM25723-RM7300	4.326	9.109	0.052	−0.884	3.938	22.74	−0.962	−1.016

“—” indicates no detected QTL under the corresponding condition. “Intervals” indicate the QTL flanking markers on the corresponding chromosome. PVE(%) represents the percentage of total phenotypic variance explained by the QTL. Add and Dom indicate the additive and dominant effects, respectively.

### Gene prediction and expression analysis

According to GRAMENE website (www.gramene.org/) and the Rice Annotation Project database (http://rapdb.dna.affrc.go.jp/)^47^, 10 open reading frames (ORFs) in the target region for *qHD7.2* between RM172 and RM22188 were predicted (Fig. 4C, S-Table 3). Four of the ORF genes contain reported functional domains, and the others are hypothetical proteins. We sequenced each of the 10 annotated genes, and only ORF1 and ORF2 had no polymorphisms in the coding domain between CSSL39 and 9311 (data not shown). Among the 10 genes, ORF7 (*LOC_Os07g49460*) with functional information is related to HD control.* LOC_Os07g49460* encodes a protein that contains a response regulator receiver domain and corresponds to the cloned HD gene *OsPRR37*; PRR37 was reported to show photoperiodic sensitivity and affects HD under long-day conditions^21^. Thus, we predicted that LOC_Os07g49460 could be a potential candidate for *qHD7.2*, and is hereafter named *HD7.2*. Furthermore, we aligned the *HD7.2* coding domain sequence (CDS) with *OsPRR37* of 9311 and Nipponbare (*japonica*). There were 10 mutations and an 8-bp deletion in the 9311 CDS compared with CSSL39; the 8-bp deletion produced premature translational termination and then led to a non-functional allele (Fig. 4D, S-Fig. 2). There were also five differences in the coding sequence between CSSL39 and Nipponbare, which led to five amino acid changes.

**Figure 5:**
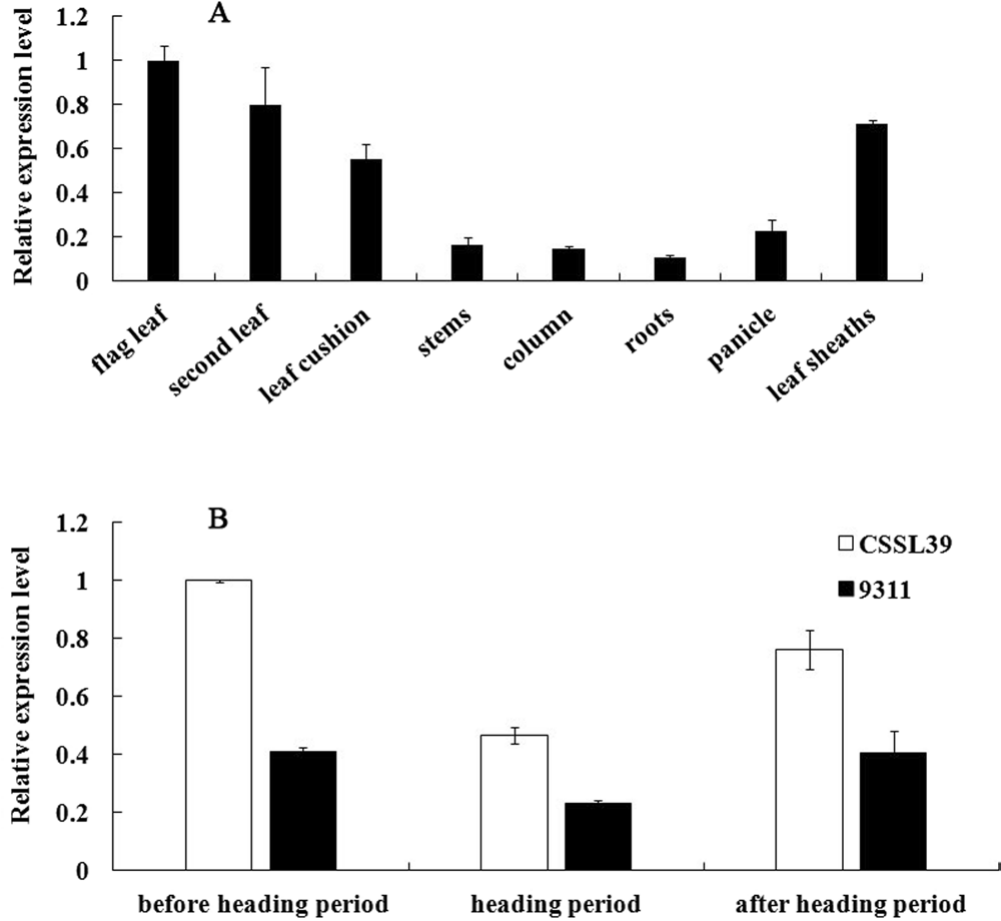
Expression analysis of *HD7.2*. (**A**) *HD7.2* expression levels in different CSSL39 tissues. (**B**) Comparison of *HD7.2/OsPRR37* transcript levels between CSSL39 and 9311 during three different stages.

The *HD7.2* spatial expression patterns were monitored in CSSL39. The *HD7.2* expression levels of the flag leaf, second leaf, leaf cushion, leaf sheaths, stems, column, roots, and panicle of CSSL39 were detected under NLD conditions in the heading period. The data show that transcript levels of this gene differed among tissues; the highest expression level was found in the flag leaf, and the lowest was in the roots (Fig. 5A). We compared the *HD7.2* expression levels between CSSL39 and 9311 in three different stages (before heading, heading period, and after heading), the transcript level was approximately two-fold higher in CSSL39 than in 9311 plants (Fig. 5B). Then, we compared the promoter region sequences that were located 2.0-kb upstream of the initiation codon between CSSL39 and 9311. The data show that there were many mutations or InDels in 9311 compared with CSSL39, and these polymorphic sites in the promoter region changed some *cis*-acting elements (S-Fig. 3); this indicated that functional proteins and the transcript level of *HD7.2/OsPRR37* resulted in later flowering.

### Nucleotide diversity analyses and haplotype network for the PRR37

**Figure 6:**
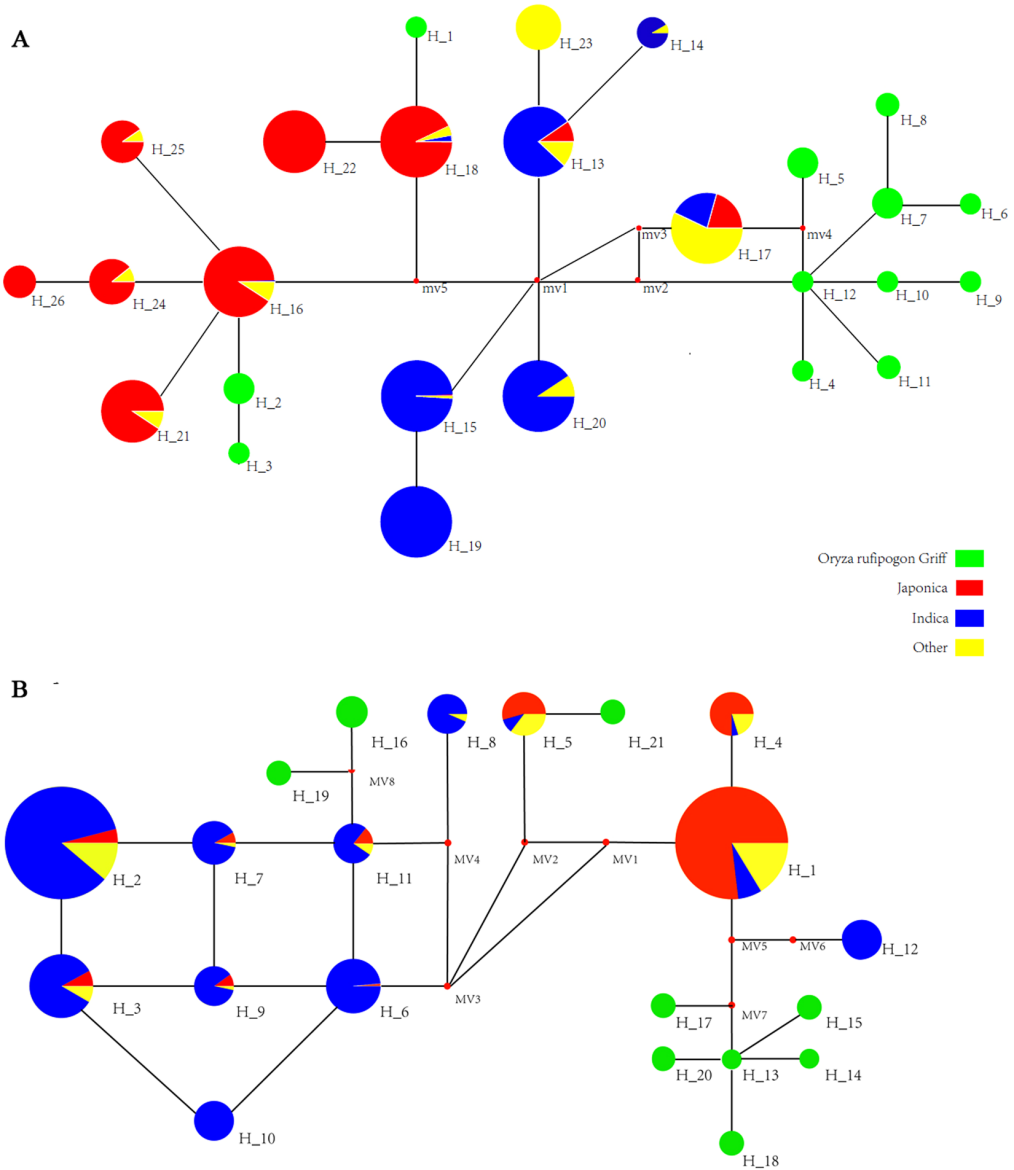
Coding (**A**) and promoter (**B**) region haplotype networks of *LOC_Os07g49460* alleles. Circle size is proportional to sample quantity within a given haplotype, and the numbers next to the circle represent haplotype number. Lines between haplotypes represent mutational steps between alleles. Colours represent different species: green, *O. rufipogon*; blue, *indica*; red, *japonica*; and yellow, others.

Using the Rice Functional Genomics-based Breeding (RFGB) Database (http://www.rmbreeding.cn/index), we aligned the PRR37 coding and promoter sequences to 2859 cultivated rice and 129 wild rice accessions. Abundant genetic variations were detected at LOC_Os07g49460 in the 2859 cultivated rice and 129 wild rice accessions, which included 44 non-synonymous single nucleotide polymorphisms (SNPs) in the coding sequence and 36 SNPs in their 2000-bp upstream promoter region. Two SNPs at position 1045 (A/C or T) and 2061 (C/T) were only found in cultivated rice; position 2061 is a synonymous SNP, whereas position 1045 is non-synonymous and corresponds to Lys/Gln (K349Q) amino acid replacement. Twenty-six haplotypes which contained more than 10 cultivated individuals or five wild rice individuals were selected, and Fig. 6A shows the network constructed with the major haplotypes for the LOC_Os07g49460 CDS; all individuals from H_1–12 were wild rice. H_1, H_2, and H_3 were closely related to *japonica* and H_4–12 was closely related to *indica*. Because the adjacent haplotypes in the network had very similar nucleotide polymorphisms (for example, H_2 and H_16 only had two SNPs at position 1045 and 2061, as mentioned above), the cultivated individuals might have evolved from the wild individuals with adjacent haplotypes. Therefore, *O. rufipogon* accessions H_1–3 were likely the direct progenitors of the *O. sativa* accessions H_16, H_18, H_21–22, and H_24–26, which are mostly *japonica*, and most of the *indica* accessions likely originated from other wild rice accessions in the network.

The network of the LOC_Os07g49460 promoter sequence were also constructed using the same database; 21 haplotypes which contain more than 10 cultivated individuals or five wild rice individuals were selected (Fig. 6B). The promoter sequence of LOC_Os07g49460 allele had high diversity in both cultivated rice and wild rice. The cultivated rice haplotypes H_1 and H_12 were most closely related to the wild rice haplotypes H_13–15, H_17–18, and H_20. More than 75% of H_1 individuals were *japonica*, and all H_12 individuals were *indica*, which indicated that both *indica* and *japonica* promoter regions originated from wild rice and be paralleled domesticated. Other cultivated rice haplotypes, including H_2–3, H_6, and H_7–11, of which most were *indica*, were closely related to the wild rice haplotypes H_16 and H_19. This finding indicates that the promoter region of different cultivated rice originated from different wild rice species, and the promoter region was also selected during domestication.

## Discussion

Previous studies showed that *O. rufipogon* from southern China is the ancestor of *O. sativa*, and many alleles in the wild species were lost during rice domestication^48,49^. The exploitation of novel alleles from wild rice that were lost in cultivated rice could be very important for rice breeding and evolution studies. Genetic populations played a major role in QTL detection and gene mapping. CSSLs have the potential to facilitate identification of QTLs not identified in F_2_ populations because of genetic background noise. In our previous study, we developed a CSSL population with a 9311 genetic background which cover the whole wild rice genome; many QTLs associated with various agronomic traits were identified using this CSSL population^46^. In this study, we identified one HD QTL, *qHD7.2*, using this CSSL population, which had the highest LOD value and has stable inheritance in all environments (Table 1). One CSSL, CSSL39, which consistently produced the latest heading date and carried the *qHD7.2* but none of other HD QTLs from the donor parent, was selected for advanced study. CSSL39 exhibited a substantially shorter panicle than 9311 under NLD and NSD conditions. Because dense SSR markers were used during the development of this CSSL population, CSSL39 was not a near isogenic line, and eight substituted segments from wild rice were detected in its genome. Furthermore, an F_2_ population of CSSL39 and recurrent parent 9311 was constructed for fine mapping of *qHD7.2*. Ten polymorphic markers located at the substituted interval around RM172 on Chr. 7 were used for additional analysis of almost 3000 F_2_ plants. Finally, a pleiotropic QTL response for HD and PL was detected between two markers: RM172 and RM22188. Theoretically, an isogenic *qHD7.2* line and F_3_ recombinants should be constructed to confirm our results. However, a previously cloned HD gene, *Os07g49460* (*OsPRR37*), was found in this interval, and an 8-bp deletion led to a nonfunctional allele. Although another PL QTL was also detected on Chr. 10, we designed InDel primers for *HD7.2* detection in F_2_ and F_3_ populations, and all individuals with the wild rice allele showed later HDs and shorter PLs (data not shown). Therefore, we can exclude other intervals, and it can be deduced that *HD7.2* is the target gene involved in shaping HD and PL phenotypes. These findings also illustrate that these CSSLs are suitable and efficient for fine mapping.

Pseudo-response regulators (PRRs) have been reported to be important circadian-clock components in *Arabidopsis* and rice^42,50^. The regulatory roles of the PRR37 orthologues in growth and development diverged among species; for example, the *Arabidopsis prr7* loss-of-function mutants flower slightly later under inductive long-day conditions, but rice *prr37*-knockout mutants flower early in non-inductive long-day conditions^21,42^. One member of rice PRR gene family, OsPRR37 is responsible for *EH7-2/Hd2*, which is the major effect QTL that controls photoperiod sensitivity in rice. OsPRR37 down-regulates *Hd3a* expression to suppress flowering under long-day conditions, and the natural variation in *OsPRR37* regulates HD and contributes to rice cultivation at a wide range of latitudes^21^. Some homologous genes of *OsPRR37* in barley, wheat, and sorghum have also been well documented to regulate flowering time^43–45^. In this study, the PRR37 allele from wild rice also substantially suppresses flowering under NLD conditions. Previous studies reported that PRR37 also affects other important agronomic traits such as plant height and spikelets per panicle^42–45^. However, no reports previously found that PRR37 affects PL, so we predicted that *HD7.2* is likely a novel allele of PRR37 that delays flowering and shortens PL under long-day conditions.

Mapping of QTLs for HD and yield component traits in rice has resulted in remarkable progress in elucidating the genetic basis that underlies the natural variation of these traits. Major QTLs for HD and yield component traits have shown a common association between delayed heading and increased yield, such as *Ghd7*^15,51^, *DTH8/Ghd8/qHY-8/LH8*^52–55^, *Hd1*^29^, and *Ghd7.1*^56^. PL is a major grain yield component trait; in this study, *HD7.2* delayed HD but shortened PL, potentially because *HD7.2* from wild rice controls the physiological process of panicle development. Our data indicated that *HD7.2* is likely a novel allele of *PRR37* that has a different function. Further studies, such as a transgenic experiment of *HD7.2*, should be performed to understand the exact function of this gene.

There were many nucleotide changes in both coding and promoter sequences of *HD7.2* between the two parents, and the expression level in CSSL39 was higher than that in 9311 during each period. In the 9311 coding sequences, an 8-bp deletion produced premature translational termination. In the promoter sequences, the changes led to *cis*-factor element differences between CSSL39 and 9311; these data indicated that the CSSL39 phenotype changes were caused by the changes of *HD7.2* transcript level and protein function. Nucleotide diversity and network analysis of PRR37 were implemented using more than 3000 cultivated and wild rice accessions. The data showed that this gene originated from multiple wild rice accessions, which is consistent with previous reports that *japonica* and *indica* evolved from multiple ancestral populations^48^. Furthermore, our result showed that the promoter region also originated from wild rice, and substantial natural variation was found in rice landraces. Koo *et al*.^21^ reported that natural variation of the PRR37 protein contributed to the wide range of latitudes in which cultivated rice can be grown; usually, *japonica*, which is distributed in high-latitude regions, has the non-functional allele^21^. In this study, some landraces had functional alleles, but the promoter was low-expression type, so they still can flowering normally under long-day conditions. It can be concluded that both the *HD7.2* coding and promoter regions were selected during domestication, and natural variation in the *PRR37* promoter region also contributed to the widespread distribution of cultivated rice. Our study could provide a novel understanding of the rice *OsPRR37* gene and rice flowering regulation networks, and provides additional evidence regarding the evolution of this gene in rice domestication.

## Materials and Methods

### Plant materials and growth conditions

A set of 198 CSSLs produced from common wild rice (*O. rufipogon*) as the donor and an elite *indica* variety, 9311, as the recurrent parent was developed in our laboratory as previously reported^42^. Each CSSL was genotyped using 313 polymorphic SSR markers evenly distributed across the 12 rice chromosomes. In this study, the CSSL population was employed for QTL mapping of HD. The genotypes of each individual were surveyed by SSR analysis; among them, one line, CSSL39, was selected as the starting material for the present study. CSSL39 was backcrossed with 9311, and the resultant F_1_ was self-crossed to produce F_2_ seeds.

The 198 CSSLs and recurrent parent 9311 were grown in six environments (two years × three locations) (S-Table 4) using the randomised complete block design with two replications. Each plot consisted of rows with 10 plants. Forty plants of each genotype in each plot were planted with a 10 × 25-cm spacing. Crop management, and disease and insect pest control were performed as locally recommended. The F_2_ CSSL39/9311 population was planted in Beijing from May–Oct 2016 and Sanya from Dec 2015–May 2016.

### Phenotype investigation

The mean value of 10 representative individual plants in the middle of the entry plot were selected for the CSSLs and 9311. HD was measured on a single-plant basis. Days to heading for each individual were scored when the first panicle (2-cm-long) emerged. PL is the length from the panicle neck to tip of the main panicle, but does not include awn length. Seed maturity percentages were measured from two panicles sampled 60 d after 9311 heading under NLD and NSD conditions, which was determined by yellow pigmentation of the seed coat.

### QTL analysis and predicted candidate gene

Initially, a total of 191 polymorphic SSR markers selected from a public database (Rice Genome Research Program 2007) were employed to construct the linkage map. To construct a high-density linkage map for fine mapping of the QTL, new InDel markers that cover the target QTL region were developed. The Nipponbare and 9311 target sequences were obtained from publicly available rice genome sequence data to develop InDel markers. Primers were designed based on InDel sequences using Primer Premier 5.0. All primer pairs flanking SSRs or InDels were designed using the following parameters: 18–25 nucleotides in length, absence of secondary structure, a GC content of approximately 50%, and a melting temperature around 55 °C. SSR and InDel marker primers were synthesised by Shanghai Invitrogen Biotechnology Company (Shanghai, China). Polymorphisms of the SSR and InDel markers between the two parents were tested by PCR. DNA of the samples was extracted from fresh leaves at the seedling stage by employing the CTAB method. PCR amplification consisted of a denaturing step of 5 min at 95 °C; followed by 33 cycles of 30 s at 94 °C, 30 s at 56 °C, and 30 s at 72 °C; finally, 10 min at 72 °C. Amplifications were separated by 6% denatured polyacrylamide gel electrophoresis and visualised by silver staining.

QTL analysis was conducted by combined the genotype with phenotype of CSSLs and secondary separation population using QTL IciMapping^57^. Mapping standard was identified as LOD⩾2.5, because a QTL exists when LOD ⩾2.5. Putative genes in the *qHD7.2* region were predicted by referring to the Rice Genome Annotation Project (http://rice.plantbiology.msu.edu/). Total RNA was extracted from leaves of the two parents using Trizol reagent (Invitrogen, CA, USA) and reversely transcribed into cDNA using a Reverse Transcription Kit (TaKaRa, Otsu, Japan). The coding regions of the putative genes were amplified from cDNA using PFU polymerase (TaKaRa, Otsu, Japan) and sequenced by Shanghai Sangon Biotechnology Company (Shanghai, China). DNA sequence comparison between the parents was performed using the BLAST program.

### Gene expression analysis

The CSSL39 plants were grown under NLD conditions for 140 d, which was before heading. The flag leaf, second leaf, leaf cushion, stems, column, roots, panicle, and leaf sheaths were harvested. All samples were harvested from the main culm of each plant. Samples from two or three different individuals were collected as biological replicates. For expression comparison, two parents were planted in the Chinese Academy of Agricultural Sciences greenhouse. Each pot in half, planting 3 pots to make each pot have CSSL39 and 9311, to ensure consistent planting conditions. Fresh leaves were collected before heading, at heading, and after heading. RNA was extracted using TRIzol Reagent (Invitrogen, CA, USA) and treated with DNase I (Invitrogen, CA, USA). cDNA was synthesised using SuperScript III Reverse Transcriptase (Invitrogen, CA, USA). Quantitative analysis of gene expression was performed with SYBR Premix Ex Taq (TaKaRa, Otsu, Japan) on an Applied Biosystems 7500 Real-time PCR System. The data were analysed using the relative quantification method.

### Network and genetic diversity analyses

We collected SNP and InDel genomic variation data for the 2859 rice genomes, and established a comprehensive SNP and InDel sub-database for the Rice Functional Genomics and Breeding Database (http://www.rmbreeding.cn/snp3k)^58^. This sub-database is a global resource that contains tools such as a polymorphism information retrieval function, genome browser visualization system, and data export system for specific genomic regions. All the SNPs located in the promoter and CDS regions were extracted based on the genome gff3 annotation. Haplotype analysis was performed using Perl scripts, and only non-synonymous SNPs were considered. Number of haplotypes and haplotypes diversity were counted by DnaSPv5 software (http://www.ub.edu/dnasp)^59^ and introduced to NETWORK 5.0.0.0 programme (Fluxus technology Ltd. 2015) for haplotype networks construction.

## Electronic supplementary material

Supplemental Fig.S1

Supplemental Fig.S2

Supplemental Fig.S3

Supplemental Table S1

Supplemental Table S2

Supplemental Table S3

Supplemental Table S4

## References

[1] Oka, H. I. *Origin of cultivated rice*. Vol. 14 (Elsevier, 2012).

[2] Khush, G. S. Origin, dispersal, cultivation and variation of rice. *Oryza: From Molecule to Plant* 25–34 (Springer, 1997). 9291957

[3] Izawa T (2007). Adaptation of flowering-time by natural and artificial selection in Arabidopsis and rice. J Exp Bot.

[4] Meyer RS, Purugganan MD (2013). Evolution of crop species: genetics of domestication and diversification. Nat Rev Genet.

[5] Monaco MK (2014). Gramene 2013: comparative plant genomics resources. Nucleic Acids Res.

[6] Kojima S (2002). *Hd3a*, a rice ortholog of the Arabidopsis *FT* gene, promotes transition to flowering downstream of *Hd1* under short-day conditions. Plant Cell Physiol.

[7] Komiya R, Yokoi S, Shimamoto K (2009). A gene network for long-day flowering activates *RFT1* encoding a mobile flowering signal in rice. Development.

[8] Yano M (2000). *Hd1*, a major photoperiod sensitivity quantitative trait locus in rice, is closely related to the Arabidopsis flowering time gene *CONSTANS*. Plant Cell.

[9] Doi K (2004). *Ehd1*, a B-type response regulator in rice, confers short-day promotion of flowering and controls *FT-like* gene expression independently of *Hd1*. Gene Dev.

[10] Zhao J (2012). *OsELF3-1*, an ortholog of arabidopsis *EARLY FLOWERING 3*, regulates rice circadian rhythm and photoperiodic flowering. PLoS One.

[11] Hayama R (2003). Adaptation of photoperiodic control pathways produces short-day flowering in rice. Nature.

[12] Andrés F (2009). Analysis of *Photoperiod Sensitivity5* sheds light on the role of phytochromes in photoperiodic flowering in rice. Plant Physiol.

[13] Ishikawa R (2011). Phytochrome B regulates *Heading date 1* (*Hd1*)-mediated expression of rice florigen *Hd3a* and critical day length in rice. Mol Gen Genet.

[14] Takahashi Y, Shomura A, Sasaki T, Yano M (2001). *Hd6*, a rice quantitative trait locus involved in photoperiod sensitivity, encodes the α subunit of protein kinase CK2. Proc Natl Acad Sci USA.

[15] Xue W (2008). Natural variation in *Ghd7* is an important regulator of heading date and yield potential in rice. Nat Genet.

[16] Matsubara K (2011). *Ehd3*, encoding a plant homeodomain finger-containing protein, is a critical promoter of rice flowering. Plant J.

[17] Peng LT (2007). Ectopic expression of *OsLFL1* in rice represses *Ehd1* by binding on its promoter. Biochem Bioph Res Co.

[18] Dai C, Xue HW (2010). Rice *early flowering1*, a CKI, phosphorylates della protein *SLR1* to negatively regulate gibberellin signalling. EMBO J.

[19] Wu C (2008). *RID1*, encoding a Cys2*/*His2-type zinc finger transcription factor, acts as a master switch from vegetative to floral development in rice. Proc Natl Acad Sci USA.

[20] Song LK (2007). *OsMADS51* is a short-day flowering promoter that functions upstream of *Ehd1*, *OsMADS14*, and *Hd3a*. Plant Physiol.

[21] Koo BH (2013). Natural variation in *OsPRR37* regulates heading date and contributes to rice cultivation at a wide range of latitudes. Mol Plant.

[22] Kim SK (2008). *OsCO3*, a *CONSTANS-LIKE* gene, controls flowering by negatively regulating the expression of *FT-like* genes under SD conditions in rice. Planta.

[23] Wu W (2013). Association of functional nucleotide polymorphisms at *DTH2* with the northward expansion of rice cultivation in Asia. Proc Natl Acad Sci USA.

[24] Li D (2009). Functional characterization of rice *OsDof12*. Planta.

[25] Takahashi Y (2009). Variations in *Hd1* proteins, *Hd3a* promoters, and *Ehd1* expression levels contribute to diversity of flowering time in cultivated rice. Proc Natl Acad Sci USA.

[26] Liu T, Shao D, Kovi MR, Xing Y (2010). Mapping and validation of quantitative trait loci for spikelets per panicle and 1,000-grain weight in rice (*Oryza sativa* L.). Theor Appl Genet.

[27] Huang X (2009). Natural variation at the *DEP1* locus enhances grain yield in rice. Nat Genet.

[28] Li S (2009). *Short panicle1* encodes a putative PTR family transporter and determines rice panicle size. Plant J.

[29] Zhang ZH (2012). Pleiotropism of the photoperiod-insensitive allele of *Hd1* on heading date, plant height and yield traits in rice. PLoS One.

[30] Yan WH (2011). A major QTL, *Ghd8*, plays pleiotropic roles in regulating grain productivity, plant height, and heading date in rice. Mol Plant.

[31] Bai X, Wu B, Xing Y (2012). Yield-related QTLs and Their Applications in Rice Genetic ImprovementF. J Integ Plant Biol.

[32] Guo L, Zhang ZH, Zhuang JY (2013). Quantitative trait loci for heading date and their relationship with genetic control of yield traits in rice (*Oryza sativa*). Rice Sci.

[33] Rahman ML (2009). High-resolution mapping of two rice brown planthopper resistance genes, *Bph20 (t)* and *Bph21 (t)*, originating from *Oryza minuta*. Theor Appl Genet.

[34] Song XJ (2007). A QTL for rice grain width and weight encodes a previously unknown RING-type E3 ubiquitin ligase. Nat Genet.

[35] Takai T (2007). Development of chromosome segment substitution lines derived from backcross between *indica* donor rice cultivar’Nona bokra’and *japonica* recipient cultivar’Koshihikari’. Breeding Sci.

[36] Yu B (2007). *TAC1*, a major quantitative trait locus controlling tiller angle in rice. Plant J.

[37] Zhou L (2009). Fine mapping of the grain chalkiness QTL *qPGWC-7* in rice (*Oryza sativa* L.). Theor Appl Genet.

[38] Zhou Y (2009). Deletion in a quantitative trait gene *qPE9-1* associated with panicle erectness improves plant architecture during rice domestication. Genetics.

[39] Mei H (2006). QTLs influencing panicle size detected in two reciprocal introgressive line (IL) populations in rice (*Oryza sativa* L.). Theor Appl Genet.

[40] Xiao J, Li J, Yuan L, Tanksley SD (1996). Identification of QTLs affecting traits of agronomic importance in a recombinant inbred population derived from a subspecific rice cross. Theor Appl Genet.

[41] Lee SJ (2005). Identification of QTLs for domestication-related and agronomic traits in an *Oryza sativa* x *O. rufipogon* BC_1_F_7_ population. Plant Breeding.

[42] Murakami M, Matsushika A, Ashikari M, Yamashino T, Mizuno T (2005). Circadian-associated rice pseudo response regulators (*OsPRRs*): insight into the control of flowering time. Biosci, Biotech Bioch.

[43] Turner A, Beales J, Faure S, Dunford RP, Laurie DA (2005). The pseudo-response regulator *Ppd-H1* provides adaptation to photoperiod in barley. Science.

[44] Beales J (2007). A pseudo-response regulator is misexpressed in the photoperiod insensitive *Ppd-D1a* mutant of wheat (*Triticum aestivum* L.). Theor Appl Genet.

[45] Murphy RL (2011). Coincident light and clock regulation of pseudo response regulator protein 37 (PRR37) controls photoperiodic flowering in sorghum. Proc Natl Acad Sci USA.

[46] Qiao W (2016). Development and characterization of chromosome segment substitution lines derived from *Oryza rufipogon* in the genetic background of *O. sativa spp. indica* cultivar 9311. BMC Genomics.

[47] Sakai H (2013). Rice Annotation Project Database (RAP-DB): an integrative and interactive database for rice genomics. Plant Cell Physiol.

[48] Huang X (2012). A map of rice genome variation reveals the origin of cultivated rice. Nature.

[49] Sun C, Wang X, Li Z, Yoshimura A, Iwata N (2001). Comparison of the genetic diversity of common wild rice (*Oryza rufipogon* Griff.) and cultivated rice (*O. sativa* L.) using RFLP markers. Theor Appl Genet.

[50] Alabadí D (2001). Reciprocal regulation between *TOC1* and *LHY*/*CCA1* within the Arabidopsis circadian clock. Science.

[51] Weng, X. *et al*. *Ghd7* is a central regulator for growth, development, adaptation and responses to biotic and abiotic stresses. *Plant Physiol*, 10.1104/pp.113.231308 (2014).

[52] Yan WH (2011). A major QTL, *Ghd8*, plays pleiotropic roles in regulating grain productivity, plant height, and heading date in rice. Mol plant.

[53] Wei X (2010). *DTH8* suppresses flowering in rice, influencing plant height and yield potential simultaneously. Plant Physiol.

[54] Cai HY (2012). Genetic and physical mapping of *qHY-8*, a pleiotropic QTL for heading date and yield-related traits in rice. Euphytica.

[55] Chen J (2014). Characterization of epistatic interaction of QTLs *LH8* and *EH3* controlling heading date in rice. Sci Rep.

[56] Yan W (2013). Natural variation in *Ghd7. 1* plays an important role in grain yield and adaptation in rice. Cell Res.

[57] Meng L (2015). QTL IciMapping: Integrated software for genetic linkage map construction and quantitative trait locus mapping in biparental populations. The Crop Journal.

[58] Zheng TQ (2015). Rice functional genomics and breeding database (RFGB): 3K-rice SNP and InDel sub-database. Chinese Science Bulletin.

[59] Librado P, Rozas J (2009). DnaSP v5: a software for comprehensive analysis of DNA polymorphism data. Bioinformatics.

