# Supplementary material for: A heading date QTL, qHD7.2, from wild rice (Oryza rufipogon) delays flowering and shortens panicle length under long-day conditions
Source: Sci Rep. 2018 Feb 13;8:2928. doi: 10.1038/s41598-018-21330-z (PMC5811536; doi:10.1038/s41598-018-21330-z)
Supplement: Supplementary file 3 — Supplemental Fig.S3 [file 41598_2018_21330_MOESM3_ESM.pdf]

A heading date QTL, *qHD7.2*, from wild rice (*Oryza rufipogon*) delays flowering and shortens panicle length under long-day conditions

Li Jing[1], Xu Rui[1],Wang Chunchao[1], Qi Lan, Zheng Xiaoming, Wang wensheng, Ding Yingbin, Zhang Lizhen, Wang Yanyan, Cheng Yunlian, Zhang Lifang, Qiao Weihua*,Yang Qingwen*

Institute of Crop Science, Chinese Academy of Agricultural Sciences, Beijing 100081, China.

[1]These authors contributed equally to this work.

*Corresponding authors:

Qiao Weihua: qiaoweihua@caas.cn; Yang Qingwen: yangqingwen@caas.cn 86-10-62186687(Tel); 86-10-62189165(Fax).

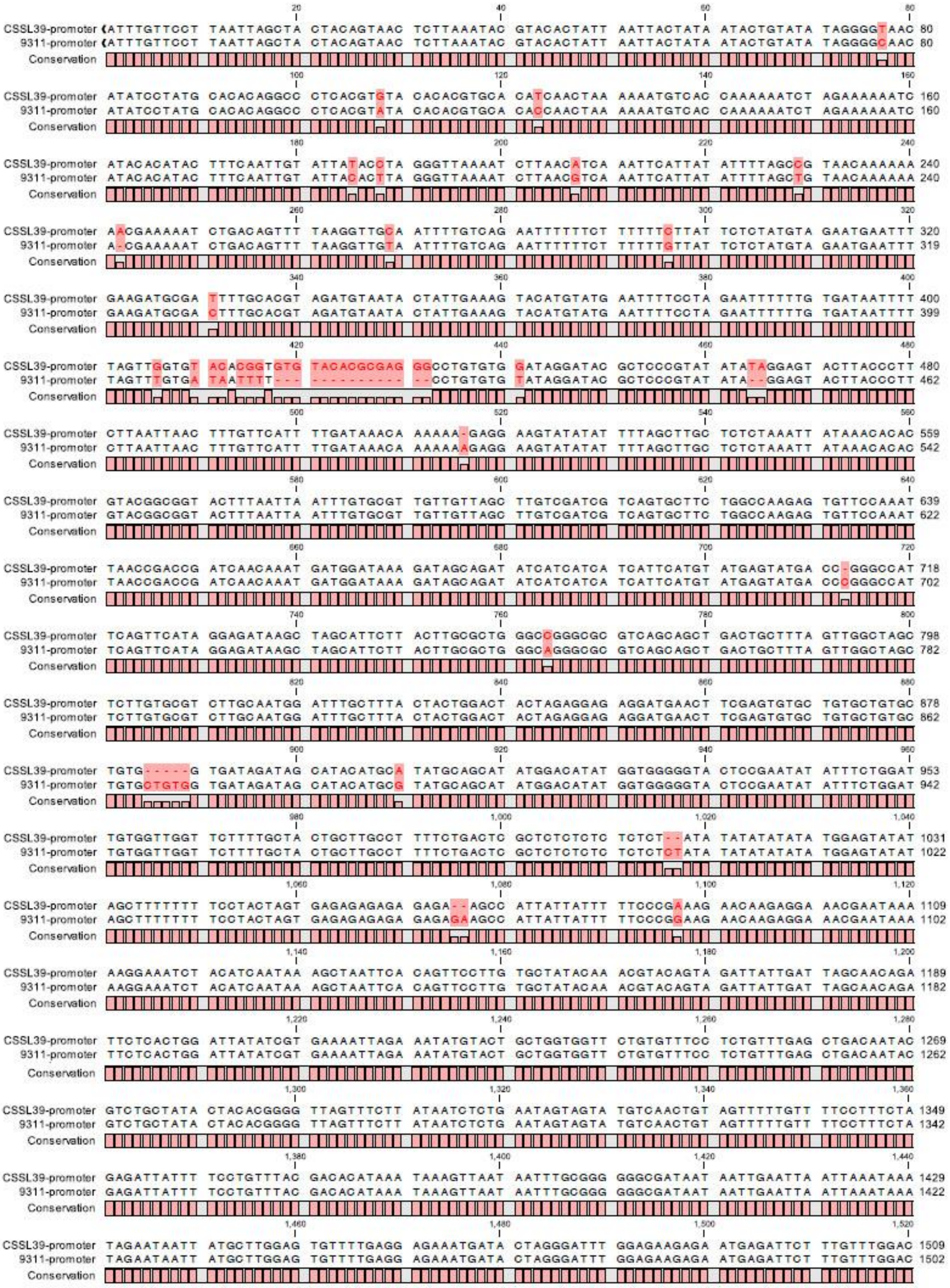
CSSL39-promoter ATTTGTTCCT TAATTAGCTA CTACAGTAAC TCTTAAATAC GTACACTATT AATTACTATA ATACTGTATA TAGGGGTAAC 80
9311-promoter ATTTGTTCCT TAATTAGCTA CTACAGTAAC TCTTAAATAC GTACACTATT AATTACTATA ATACTGTATA TAGGGGCAAC 80
Conservation
CSSL39-promoter ATATCCTATG CACACAGGCC CTCACGTGTA CACACGTGCA CATCAACTAA AAAATGTCAC CAAAAAATCT AGAAAAAATC 160
9311-promoter ATATCCTATG CACACAGGCC CTCACGTATA CACACGTGCA CACCAACTAA AAAATGTCAC CAAAAAATCT AGAAAAAATC 160
Conservation
CSSL39-promoter ATACACATAC TTTCAATTGT ATTATACCTA GGGTTAAAAT CTTAACATCA AATTCATTAT ATTTTAGCCG TAACAAAAAA 240
9311-promoter ATACACATAC TTTCAATTGT ATTACACTTA GGGTTAAAAT CTTAACGTCA AATTCATTAT ATTTTAGCTG TAACAAAAAA 240
Conservation
CSSL39-promoter AACGAAAAAT CTGACAGTTT TAAGGTTGCA ATTTTGTCAG AATTTTTTCT TTTTTCTTAT TCTCTATGTA GAATGAATTT 320
9311-promoter A-CGAAAAAT CTGACAGTTT TAAGGTTGTA ATTTTGTCAG AATTTTTTCT TTTTTGTTAT TCTCTATGTA GAATGAATTT 319
Conservation
CSSL39-promoter GAAGATGCGA TTTTGCACGT AGATGTAATA CTATTGAAAG TACATGTATG AATTTTCCTA GAATTTTTTG TGATAATTTT 400
9311-promoter GAAGATGCGA CTTTGCACGT AGATGTAATA CTATTGAAAG TACATGTATG AATTTTCCTA GAATTTTTTG TGATAATTTT 399
Conservation
CSSL39-promoter TAGTTGGTGT ACACGGTGTG TACACGCGAG GGCCTGTGTG GATAGGATAC GCTCCCGTAT ATATAGGAGT ACTTACCCTT 480
9311-promoter TAGTTTGTGA TAATTTT--- ---------- --CCTGTGTG TATAGGATAC GCTCCCGTAT ATA--GGAGT ACTTACCCTT 462
Conservation
CSSL39-promoter CTTAATTAAC TTTGTTCATT TTGATAAACA AAAAA-GAGG AAGTATATAT TTTAGCTTGC TCTCTAAATT ATAAACACAC 559
9311-promoter CTTAATTAAC TTTGTTCATT TTGATAAACA AAAAAAGAGG AAGTATATAT TTTAGCTTGC TCTCTAAATT ATAAACACAC 542
Conservation
CSSL39-promoter GTACGGCGGT ACTTTAATTA ATTTGTGCGT TGTTGTTAGC TTGTCGATCG TCAGTGCTTC TGGCCAAGAG TGTTCCAAAT 639
9311-promoter GTACGGCGGT ACTTTAATTA ATTTGTGCGT TGTTGTTAGC TTGTCGATCG TCAGTGCTTC TGGCCAAGAG TGTTCCAAAT 622
Conservation
CSSL39-promoter TAACCGACCG ATCAACAAAT GATGGATAAA GATAGCAGAT ATCATCATCA TCATTCATGT ATGAGTATGA CC-GGGCCAT 718
9311-promoter TAACCGACCG ATCAACAAAT GATGGATAAA GATAGCAGAT ATCATCATCA TCATTCATGT ATGAGTATGA CCCGGGCCAT 702
Conservation
CSSL39-promoter TCAGTTCATA GGAGATAAGC TAGCATTCTT ACTTGCGCTG GGCCGGGCGC GTCAGCAGCT GACTGCTTTA GTTGGCTAGC 798
9311-promoter TCAGTTCATA GGAGATAAGC TAGCATTCTT ACTTGCGCTG GGCAGGGCGC GTCAGCAGCT GACTGCTTTA GTTGGCTAGC 782
Conservation
CSSL39-promoter TCTTGTGCGT CTTGCAATGG ATTTGCTTTA CTACTGGACT ACTAGAGGAG AGGATGAACT TCGAGTGTGC TGTGCTGTGC 878
9311-promoter TCTTGTGCGT CTTGCAATGG ATTTGCTTTA CTACTGGACT ACTAGAGGAG AGGATGAACT TCGAGTGTGC TGTGCTGTGC 862
Conservation
CSSL39-promoter TGTG-----G TGATAGATAG CATACATGCA TATGCAGCAT ATGGACATAT GGTGGGGGTA CTCCGAATAT ATTTCTGGAT 953
9311-promoter TGTGCTGTGG TGATAGATAG CATACATGCG TATGCAGCAT ATGGACATAT GGTGGGGGTA CTCCGAATAT ATTTCTGGAT 942
Conservation
CSSL39-promoter TGTGGTTGGT TCTTTTGCTA CTGCTTGCCT TTTCTGACTC GCTCTCTCTC TCTCT--ATA TATATATATA TGGAGTATAT 1031
9311-promoter TGTGGTTGGT TCTTTTGCTA CTGCTTGCCT TTTCTGACTC GCTCTCTCTC TCTCTCTATA TATATATATA TGGAGTATAT 1022
Conservation
CSSL39-promoter AGCTTTTTTT TCCTACTAGT GAGAGAGAGA GAGA--AGCC ATTATTATTT TTCCCGAAAG AACAAGAGGA AACGAATAAA 1109
9311-promoter AGCTTTTTTT TCCTACTAGT GAGAGAGAGA GAGAGAAGCC ATTATTATTT TTCCCGGAAG AACAAGAGGA AACGAATAAA 1102
Conservation
CSSL39-promoter AAGGAAATCT ACATCAATAA AGCTAATTCA CAGTTCCTTG TGCTATACAA ACGTACAGTA GATTATTGAT TAGCAACAGA 1189
9311-promoter AAGGAAATCT ACATCAATAA AGCTAATTCA CAGTTCCTTG TGCTATACAA ACGTACAGTA GATTATTGAT TAGCAACAGA 1182
Conservation
CSSL39-promoter TTCTCACTGG ATTATATCGT GAAAATTAGA AATATGTACT GCTGGTGGTT CTGTGTTTCC TCTGTTTGAG CTGACAATAC 1269
9311-promoter TTCTCACTGG ATTATATCGT GAAAATTAGA AATATGTACT GCTGGTGGTT CTGTGTTTCC TCTGTTTGAG CTGACAATAC 1262
Conservation
CSSL39-promoter GTCTGCTATA CTACACGGGG TTAGTTTCTT ATAATCTCTG AATAGTAGTA TGTCAACTGT AGTTTTTGTT TTCCTTTCTA 1349
9311-promoter GTCTGCTATA CTACACGGGG TTAGTTTCTT ATAATCTCTG AATAGTAGTA TGTCAACTGT AGTTTTTGTT TTCCTTTCTA 1342
Conservation
CSSL39-promoter GAGATTATTT TCCTGTTTAC GACACATAAA TAAAGTTAAT AATTTGCGGG GGGCGATAAT AATTGAATTA ATTAAATAAA 1429
9311-promoter GAGATTATTT TCCTGTTTAC GACACATAAA TAAAGTTAAT AATTTGCGGG GGGCGATAAT AATTGAATTA ATTAAATAAA 1422
Conservation
CSSL39-promoter TAGAATAATT ATGCTTGGAG TGTTTTGAGG AGAAATGATA CTAGGGATTT GGAGAAGAGA ATGAGATTCT TTGTTTGGAC 1509
9311-promoter TAGAATAATT ATGCTTGGAG TGTTTTGAGG AGAAATGATA CTAGGGATTT GGAGAAGAGA ATGAGATTCT TTGTTTGGAC 1502
Conservation

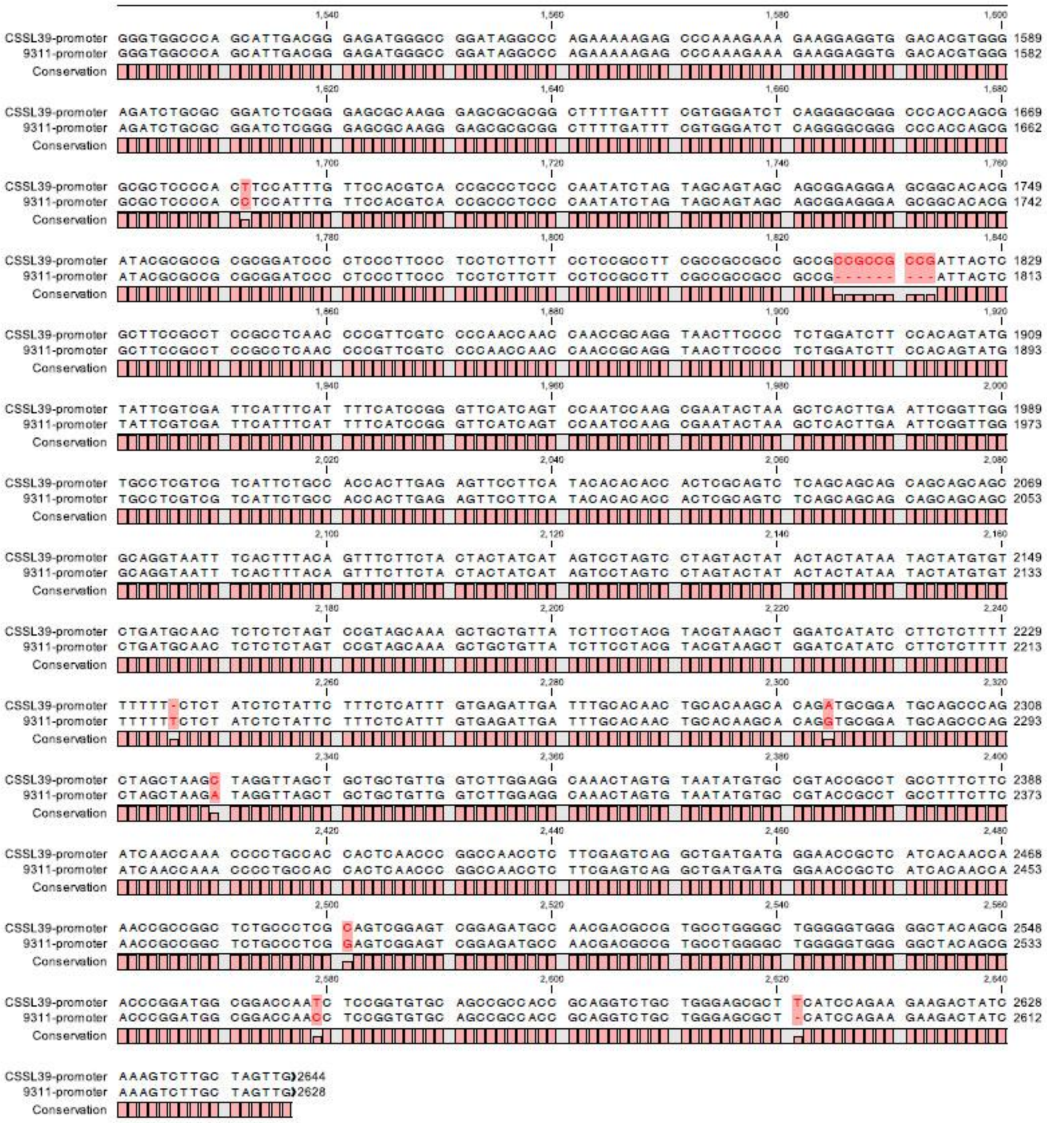

Supplemental Fig.S3. Alignment of LOC_Os07g49460 promoter sequences of CSSL39 and 9311; SNPs are indicated in red.
