# Supplementary material for: A heading date QTL, qHD7.2, from wild rice (Oryza rufipogon) delays flowering and shortens panicle length under long-day conditions
Source: Sci Rep. 2018 Feb 13;8:2928. doi: 10.1038/s41598-018-21330-z (PMC5811536; doi:10.1038/s41598-018-21330-z)
Supplement: Supplementary file 4 — Supplemental Table S1 [file 41598_2018_21330_MOESM4_ESM.pdf]

A heading date QTL, *qHD7.2*, from wild rice (*Oryza rufipogon*) delays flowering and shortens panicle length under long-day conditions

Li Jing[1], Xu Rui[1],Wang Chunchao[1], Qi Lan, Zheng Xiaoming, Wang wensheng, Ding Yingbin, Zhang Lizhen, Wang Yanyan, Cheng Yunlian, Zhang Lifang, Qiao Weihua*,Yang Qingwen*

Institute of Crop Science, Chinese Academy of Agricultural Sciences, Beijing 100081, China.

[1]These authors contributed equally to this work.

*Corresponding authors:

Qiao Weihua: qiaoweihua@caas.cn; Yang Qingwen: yangqingwen@caas.cn 86-10-62186687(Tel); 86-10-62189165(Fax).

S-Table 1. Statistics for nine agronomic traits of 9311 and CSSL39 populations in six environments. All data are given as mean ± SD. Each P value is obtained from a t test between CSSL39 and 9311. The ñ-ò means data has not observed because of the unripe seeds, ñ**òmeans significant differences (P<0.01). TGW: thousand grain weight; GL: grain length, GW: grain width; PH: plant height; FLL: flag leaf length; FLW: flag leaf width; and TN: tiller number.

		TGW	GL	GW	GL/GW	PH	FLL	FLW	PL	TN
	CSSL39	28.56 ± 0.7	9.16 ± 0.04	2.80 ± 0.01	3.29 ± 0.03	92.90 ± 2.15	23.98 ± 2.55	1.80 ± 0.12	17.57± 2.03	7.40 ± 1.14
2014 Sanya	9311	30.11±2.11	9.58±0.36	2.88±0.09	3.40±0.02	97.65±3.03	25.31±3.29	1.75 ± 0.07	20.83± 0.77	7.87± 1.37
	P	0.071	0.316	0.297	0.955	0.033	<0.01	0.407	<0.0001	0.090
	CSSL39	28.91 ± 3.26	9.53 ± 0.16	2.83±0.09	3.39 ± 0.05	94.06 ± 4.50	18.96 ± 3.67	2.02 ± 0.15	17.48 ± 1.11	7.80 ± 1.64
2015 Sanya	9311	29.48±2.11	9.57±0.42	2.85±0.09	3.42±0.01	98.20±4.62	23.03±4.75	1.98 ± 0.19	21.38± 0.67	7.85± 1.28
	P	0.404	0.45	0.828	0.512	0.065	0.054	0.567	<0.0001	0.31
	CSSL39	28.89 ± 0.43	10.03 ± 0.07	2.85±0.01	3.53 ± 0.02	127.5± 3.64	29.4 ± 3.30	2.14 ± 0.09	20.94 ± 1.73	8.20 ± 0.45
2014 Nanjing	9311	32.36±1.73	10.09±0.50	2.84±0.01	3.44±0.02	127.1±1.24	33.1±2.8	2.24 ± 0.05	22.93 ± 1.05	7.60± 1.45
	P	<0.01	0.668	0.233	0.394	0.064	0.565	<0.01	0.057	0.139
	CSSL39	28.11 ± 0.95	9.5 ± 0.06	2.92 ± 0.01	3.27 ± 0.02	110.7 ± 2.79	27.54 ± 5.00	2.56 ± 0.18	20.46 ± 1.35	7.64 ± 2.61
2015 Nanjing	9311	31.35±1.93	9.77±0.42	2.88±0.10	3.45±0.28	123.3±5.86	31.58±3.32	2.51±0.03	22.5 ± 0.58	7.67± 1.04
	P	0.072	0.396	0.035	0.086	<0.01	0.034	0.818	0.063	0.073
	CSSL39	-	-	-	-	94.94 ± 4.92	25.66 ± 2.47	2.32 ± 0.04	19.38 ± 0.63	7.87 ± 3.65
2014 Beijing	9311					98.99±5.77	25.73 ± 3.67	2.50± 0.03	25.67±2.58	7.79± 2.55
	P	-	-	-	-	0.132	0.793	<0.01	<0.0001	0.177
	CSSL39	-	-	-	-	79.7 ± 5.09	23.78 ± 4.48	1.98 ± 0.19	18.34 ± 0.81	7.66 ± 0.89
2015 Beijing	9311					97.86±4.97	23.67 ± 3.28	2.05± 0.11	25.09±1.97	7.90± 0.070
	P	-	-	-	-	<0.01	0.037	0.128	<0.0001	0.028
