# Supplementary material for: A heading date QTL, qHD7.2, from wild rice (Oryza rufipogon) delays flowering and shortens panicle length under long-day conditions
Source: Sci Rep. 2018 Feb 13;8:2928. doi: 10.1038/s41598-018-21330-z (PMC5811536; doi:10.1038/s41598-018-21330-z)
Supplement: Supplementary file 5 — Supplemental Table S2 [file 41598_2018_21330_MOESM5_ESM.pdf]

A heading date QTL, *qHD7.2*, from wild rice (*Oryza rufipogon*) delays flowering and shortens panicle length under long-day conditions

Li Jing[1], Xu Rui[1],Wang Chunchao[1], Qi Lan, Zheng Xiaoming, Wang wensheng, Ding Yingbin, Zhang Lizhen, Wang Yanyan, Cheng Yunlian, Zhang Lifang, Qiao Weihua*,Yang Qingwen*

Institute of Crop Science, Chinese Academy of Agricultural Sciences, Beijing 100081, China.

[1]These authors contributed equally to this work.

*Corresponding authors:

Qiao Weihua: qiaoweihua@caas.cn; Yang Qingwen: yangqingwen@caas.cn 86-10-62186687(Tel); 86-10-62189165(Fax).

S-Table 2. SSR and InDel markers used in this study.

Marker	Chr.	position	Forword sequence	Reverse sequence
RM2715	7	28647219-28647504	TGCATGTTGTACGTGTAGTGAACACC	CGCAGAGGACACCGTACACTAGC
RM7601	7	29039553-29039685	GTTTGTTGCGTCGAATGGAAAGC	CGGCTTGTGAATTTGCTTGTGG
RM22188	7	29663269-29663428	TTTGGAGGCTTTCTCGCTTTCG	GTCGAGGATGAGGAAGGCATCG
Indel7-7	7	15864712-15864610	CACTCCACAGACATGCAATTT	ATCGGTGCCGCTCCTAGAT
Indel7-8	7	18365272-18365151	TGGCTTTAGGTTTTTGTGCAT	CCCGCTGATAAGTCCAGGTA
Indel7-9	7	20884620-20884517	TCCCTCCTTGTCGTCTTTTT	TGAATTATTAGCACGCGGTTT
Indel7-10	7	23402776-23402633	TTCGTTAATCCTCACGCAGA	TCCGGCGAGAAAATAAGTGT
Indel7-11	7	25931707-25931558	GTTTTTGCGCTTTTGTTGCT	GCCCACCTGTCATTGAGAGTA
Indel7-12	7	28469729-28469617	GATCGGAGGCTTTTGTTTGA	CGTTCGTGTTTTTCGCTGAT
Indel7-13	7	29663415-29663289	GATCGGAGGCTTTTGTTTGA	TGGAGGCTTTCTCGCTTTC
