# Supplementary material for: A heading date QTL, qHD7.2, from wild rice (Oryza rufipogon) delays flowering and shortens panicle length under long-day conditions
Source: Sci Rep. 2018 Feb 13;8:2928. doi: 10.1038/s41598-018-21330-z (PMC5811536; doi:10.1038/s41598-018-21330-z)
Supplement: Supplementary file 6 — Supplemental Table S3 [file 41598_2018_21330_MOESM6_ESM.pdf]

A heading date QTL, *qHD7.2*, from wild rice (*Oryza rufipogon*) delays flowering and shortens panicle length under long-day conditions

Li Jing[1], Xu Rui[1],Wang Chunchao[1], Qi Lan, Zheng Xiaoming, Wang wensheng, Ding Yingbin, Zhang Lizhen, Wang Yanyan, Cheng Yunlian, Zhang Lifang, Qiao Weihua*,Yang Qingwen*

Institute of Crop Science, Chinese Academy of Agricultural Sciences, Beijing 100081, China.

[1]These authors contributed equally to this work.

*Corresponding authors:

Qiao Weihua: qiaoweihua@caas.cn; Yang Qingwen: yangqingwen@caas.cn 86-10-62186687(Tel); 86-10-62189165(Fax).

S-Table 3. Annotation information of candidate genes for *qHD7.2*.

**Predicted gene**	**Locus ID**	**Predicted function**
ORF1	LOC_Os07g49360	peroxidase precursor, putative, expressed
ORF2	LOC_Os07g49370	glycosyltransferase family 43 protein, putative, expressed
ORF3	LOC_Os07g49380	PWWP domain containing protein, expressed
ORF4	LOC_Os07g49390	P-protein, putative, expressed
ORF5	LOC_Os07g49400	OsAPx2 - Cytosolic Ascorbate Peroxidase encoding gene 4,5,6,8, expressed
ORF6	LOC_Os07g49410	uncharacterized ACR, YagE family COG1723 containing protein, expressed
ORF7	LOC_Os07g49460	response regulator receiver domain containing protein, expressed
ORF8	LOC_Os07g49470	protein kinase APK1B, chloroplast precursor, putative, expressed
ORF9	LOC_Os07g49480	KIP1, putative, expressed
ORF10	LOC_Os07g49520	2-oxoglutarate dehydrogenase E1 component, mitochondrial precursor, putative, expressed
