# Supplementary material for: A heading date QTL, qHD7.2, from wild rice (Oryza rufipogon) delays flowering and shortens panicle length under long-day conditions
Source: Sci Rep. 2018 Feb 13;8:2928. doi: 10.1038/s41598-018-21330-z (PMC5811536; doi:10.1038/s41598-018-21330-z)
Supplement: Supplementary file 7 — Supplemental Table S4 [file 41598_2018_21330_MOESM7_ESM.pdf]

A heading date QTL, *qHD7.2*, from wild rice (*Oryza rufipogon*) delays flowering and shortens panicle length under long-day conditions

Li Jing[1], Xu Rui[1],Wang Chunchao[1], Qi Lan, Zheng Xiaoming, Wang wensheng, Ding Yingbin, Zhang Lizhen, Wang Yanyan, Cheng Yunlian, Zhang Lifang, Qiao Weihua*,Yang Qingwen*

Institute of Crop Science, Chinese Academy of Agricultural Sciences, Beijing 100081, China.

[1]These authors contributed equally to this work.

*Corresponding authors:

Qiao Weihua: qiaoweihua@caas.cn; Yang Qingwen: yangqingwen@caas.cn 86-10-62186687(Tel); 86-10-62189165(Fax).

S-Table 4. Test environments in which the CSSL populations were evaluated.

Environment	Replication	Crop location	Cropping season
E1	2	Changping, Beijing N40.20°, E115.51°	May-Oct 2014
E2	2	Nanjing, Jiangsu N32.03°, E118.47°	May-Oct 2014
E3	2	Sanya, Hainan N18.15°, E109.3°	Dec2013-May 2014
E4	2	Changping, Beijing N40.20°, E115.51°	May-Oct 2015
E5	2	Nanjing, Jiangsu N32.03°, E118.47°	May-Oct 2015
E6	2	Sanya, Hainan N18.15°, E109.3°	Dec2014-May 2015
